# Non-invasive brain stimulation combined with three rehabilitation approaches for cognitive and emotional well-being in Parkinson’s patients

**DOI:** 10.3389/fneur.2025.1670778

**Published:** 2025-10-14

**Authors:** Qianqian Fan, Xinyu Lin, Haojie Li, Lan Li

**Affiliations:** ^1^Woosuk University, Wanju-gun, Republic of Korea; ^2^Shanghai University of Sport, Shanghai, China

**Keywords:** non-invasive brain stimulation, cognitive rehabilitation, Parkinson’s disease, emotional well-being, motor therapy

## Abstract

**Background:**

Parkinson’s disease progressively impairs both motor and non-motor functions, with over 60% of patients developing cognitive decline and nearly half suffering from depression or anxiety. While dopaminergic therapies inadequately address these symptoms, traditional rehabilitation shows inconsistent results due to impaired neuroplasticity. Non-invasive brain stimulation (tDCS/rTMS) may enhance rehabilitation by modulating neural activity, but the optimal combined approaches remain unclear. This study evaluates three rehabilitation strategies paired with brain stimulation to improve cognitive and emotional outcomes in Parkinson’s patients.

**Methods:**

We systematically searched PubMed, Web of Science, EMBASE, Cochrane Library, and China National Knowledge Infrastructure (CNKI), ultimately including 7 randomized controlled trials (15 interventions, *N* = 325 Parkinson’s patients). Outcomes assessed cognitive function and emotional well-being measures. Using STATA 18.0, we conducted a network meta-analysis to evaluate relative intervention effects and assess consistency between direct/indirect evidence. Results visualized through network plots and ranked by SUCRA probabilities.

**Results:**

The analysis revealed that cognitive rehabilitation combined with non-invasive brain stimulation (CR) showed superior efficacy for cognitive improvement (SMD = 4.88, 95% CI [−1.91, 11.67]; SUCRA = 81.2), while combined motor-cognitive rehabilitation (MCR) excelled in emotional well-being (SMD = 4.76, 95% CI [2.70, 6.82], *p* < 0.00001; SUCRA = 99.5). CR for cognitive benefits and MCR for emotional regulation, with CR demonstrating the most stable treatment effects.

**Conclusion:**

This study demonstrates that non-invasive brain stimulation combined with cognitive rehabilitation (CR) is the most effective approach for improving cognitive function in Parkinson’s patients, while combined motor-cognitive rehabilitation (MCR) shows particular efficacy for emotional well-being. The findings support personalized intervention strategies: CR for cognitive impairment and MCR for emotional symptoms. Future research should optimize combined protocols to enhance synergistic effects while minimizing patient burden. This evidence-based recommendation provides important guidance for clinical practice in managing Parkinson’s non-motor symptoms.

## 1 Introduction

Parkinson’s disease (PD) is a neurodegenerative disorder characterized by motor dysfunction as its core clinical feature (1). However, recent studies have demonstrated that the pathophysiological impact of cognitive and emotional impairments is equally significant, posing critical challenges to patients’ quality of life and disease prognosis (2). Epidemiological data indicate that approximately 60–80% of PD patients will develop mild cognitive impairment, with 50% progressing to Parkinson’s disease dementia within 5 years (3). Concurrently, the comorbidity rates of depression and anxiety disorders reach as high as 40–50% (4). These symptoms often emerge in the premotor stage and independently predict faster disease progression and higher risks of care dependency (5).

Current clinical management faces a dual dilemma: on one hand, dopaminergic medications exhibit limited efficacy in alleviating cognitive and emotional symptoms (6); on the other hand, traditional rehabilitation interventions (such as cognitive training, motor therapy, and combined motor-cognitive therapy programs) show significant heterogeneity in treatment outcomes. This variability may stem from structural degeneration in the prefrontal-striatal circuits and limbic system in PD patients, which impairs neuroplasticity (7, 8), thereby reducing the brain’s adaptive response to behavioral interventions.

Non-invasive brain stimulation (NIBS) techniques, particularly transcranial direct current stimulation (tDCS) and repetitive transcranial magnetic stimulation (rTMS), offer promising solutions to these challenges. tDCS targeting the left dorsolateral prefrontal cortex (DLPFC) can enhance executive function by modulating cortical excitability (9), while rTMS applied to the limbic system effectively alleviates emotional dysregulation (10). The synergistic application of NIBS and rehabilitation training may induce long-term potentiation-like plasticity changes, yielding additive therapeutic effects (11).

Nevertheless, systematic evaluations of the efficacy differences among various NIBS-rehabilitation combinations are lacking, and optimal intervention protocols may be symptom-specific. To address this gap, this study aims to employ a network meta-analysis (NMA) to quantitatively compare the therapeutic advantages of three rehabilitation methods combined with NIBS, providing graded recommendations for the therapeutic possibility of non-motor symptoms in PD.

## 2 Methods

### 2.1 Search strategy

This research protocol has been registered for meta-analysis on the international prospective systematic review registration platform PROSPERO, with registration number CRD420251106486.

This study systematically searched eight electronic databases (PubMed, MEDLINE, EMBASE, Web of Science, Cochrane Library, CNKI, and Wanfang Database) from their inception to May 21, 2025. A search strategy combining subject headings and free-text terms was employed, including:(1) Disease-related terms: “Parkinson Disease,” “Parkinsonian Disorders,” “Primary Parkinsonism,” “Parkinson’s disease,” “PD” (2) Intervention-related terms: “Transcranial Magnetic Stimulation,” “Transcranial Direct Current Stimulation,” “Deep Transcranial Magnetic Stimulation,” “Theta Burst Stimulation,” “Transcranial Electrical Stimulation,” “Transcranial Alternating Current Stimulation,” “Transcranial Random Noise Stimulation,” “Transcranial Ultrasound Stimulation,” “Low-Intensity Pulsed Ultrasound,” “TMS,” “rTMS,” “dTMS,” “TBS,” “tDCS,” “tACS,” “tRNS,” “TUS,” (3) Cognitive/affective terms: “Cognition,” “Cognitive Dysfunction,” “Executive Function,” “Memory,” “Attention,” “Emotional Regulation,” “Affective Symptoms,” “Mood Disorders,” “Depressive Disorder,” “Major Depressive Disorder,” “Anxiety Disorders,” “Generalized Anxiety Disorder,” “Nervousness,” “Emotional Disorder,” “Depression,” and “Anxiety.” (4) Rehabilitation-related terms:"Cognitive Training,” “Dual-Task Training,” “Psychosocial Intervention,” “Cognitive Rehabilitation,” “Cognitive Therapy,” “Motor therapy,” “Physical Therapy,” “Behavioral Therapy.”

Citation tracking (forward and backward) was performed through Web of Science, and reference lists of included studies and relevant systematic reviews were manually screened. When necessary, field experts were contacted to obtain unpublished research data.

### 2.2 Inclusion and exclusion criteria

#### 2.2.1 Inclusion criteria

*Participants*: Adult patients (age ≥ 18 years) with diagnosed Parkinson’s disease (any Hoehn-Yahr stage I–V); no restrictions on disease duration.
*Interventions*: Clear use of non-invasive brain stimulation (tDCS or rTMS) combined with rehabilitation training; specified stimulation parameters (including stimulation site, intensity, frequency, duration); detailed rehabilitation protocol (including training content, frequency, intensity).
*Control groups*: Active controls (e.g., sham stimulation combined with rehabilitation) or passive controls (e.g., rehabilitation alone or waitlist).
*Outcome measures*: Cognitive function and negative emotional symptoms.
*Study design*: Randomized controlled trials (RCTs).
*Language*: English or Chinese only.

#### 2.2.2 Exclusion criteria

Participants: Comorbid other neurological disorders (e.g., stroke, Alzheimer’s disease); severe cognitive impairment (MMSE < 24); implanted devices such as deep brain stimulation (DBS).

*Interventions*: Pharmacological interventions alone; unspecified stimulation parameters or rehabilitation protocols; combined with other invasive therapies.
*Study design*: Non-randomized controlled studies (e.g., case reports, cross-sectional studies); sample size < 10; duplicate publications.
*Others*: No control group; non-standardized outcome measures; unavailable full-text or key data.

### 2.3 Literature screening and data extraction

Literature screening was performed using EndNote X9 software for reference management and deduplication. The screening process consisted of two phases: In the first phase, two researchers independently conducted preliminary screening based on titles and abstracts to exclude studies that clearly did not meet the inclusion criteria. In the second phase, full-text articles of the initially retained studies were obtained for detailed evaluation to determine final inclusion. Any disagreements between researchers were resolved through discussion or consultation with a third researcher.

Data extraction was performed using standardized forms, including:

1. Participants: Adult patients with Parkinson’s disease;
2. Interventions: Non-invasive brain stimulation combined with cognitive rehabilitation, non-invasive brain stimulation combined with exercise rehabilitation, and non-invasive brain stimulation combined with motor-cognitive rehabilitation;
3. Comparisons: Control types and specific implementation protocols;
4. Outcomes: Cognitive function and negative emotional symptoms; and
5. Study design: First author, publication year, country/region, and study type.

Two researchers independently extracted data, followed by cross-checking. Any inconsistencies were resolved by re-examining the original articles or contacting the original authors, ensuring data extraction consistency >95%.

### 2.4 Risk of bias assessment

The Cochrane Risk of Bias Tool (RoB 2.0) was used to evaluate the methodological quality of included studies. Two researchers independently assessed each study and categorized them into three levels for each evaluation item: “low risk,” “some concerns,” or “high risk.” Disagreements were resolved through discussion or consultation with a third researcher. The final assessment results were visually presented using risk of bias summary plots, and sensitivity analyses were performed for studies with high risk of bias to ensure the robustness of study conclusions.

### 2.5 Statistical analysis

Network meta-analysis (NMA) was performed using STATA 18.0 software to systematically evaluate the intervention effects of different non-invasive brain stimulation (tDCS/rTMS) combined rehabilitation approaches on cognitive and emotional functions in Parkinson’s disease patients. All outcome measures were continuous variables, and standardized mean differences (SMD) with 95% confidence intervals (CI) were calculated to quantify intervention effects, with the significance level set at *α* = 0.05. Considering differences in patient characteristics and intervention protocols across studies, random-effects models were used to pool effect sizes, while I^2^ statistics and Cochran’s Q test were employed to assess heterogeneity.

Network plots were constructed to visually display direct comparison relationships among interventions, with node sizes representing study sample sizes and line thickness reflecting comparison frequencies. Contribution plots were also generated to quantify each direct comparison’s contribution to the entire network. To assess publication bias, adjusted funnel plots were created for primary outcomes (cognitive function scores, emotional symptom scores). Finally, the surface under the cumulative ranking curve (SUCRA) method was used to calculate the probability of each intervention combination being the optimal treatment, providing evidence-based support for clinical decision-making.

## 3 Results

### 3.1 Synthesis of studies from systematic search

This study systematically searched six databases: The Cochrane Library (*n* = 33), Embase (*n* = 87), Web of Science (*n* = 53), PubMed (*n* = 72), EBSCO (*n* = 69), and China National Knowledge Infrastructure (CNKI) (*n* = 25), yielding 339 potentially relevant records. Through rigorous screening procedures, 114 duplicate records were first removed, followed by exclusion of ineligible studies based on PICOS criteria: studies with inappropriate populations (*n* = 86), non-randomized controlled trials (*n* = 35), and mismatched interventions (*n* = 70). During full-text assessment, additional exclusions were made for studies with incompatible outcome measures (*n* = 9), inappropriate control settings (*n* = 16), and duplicate publications (*n* = 1).

**Figure 1:**
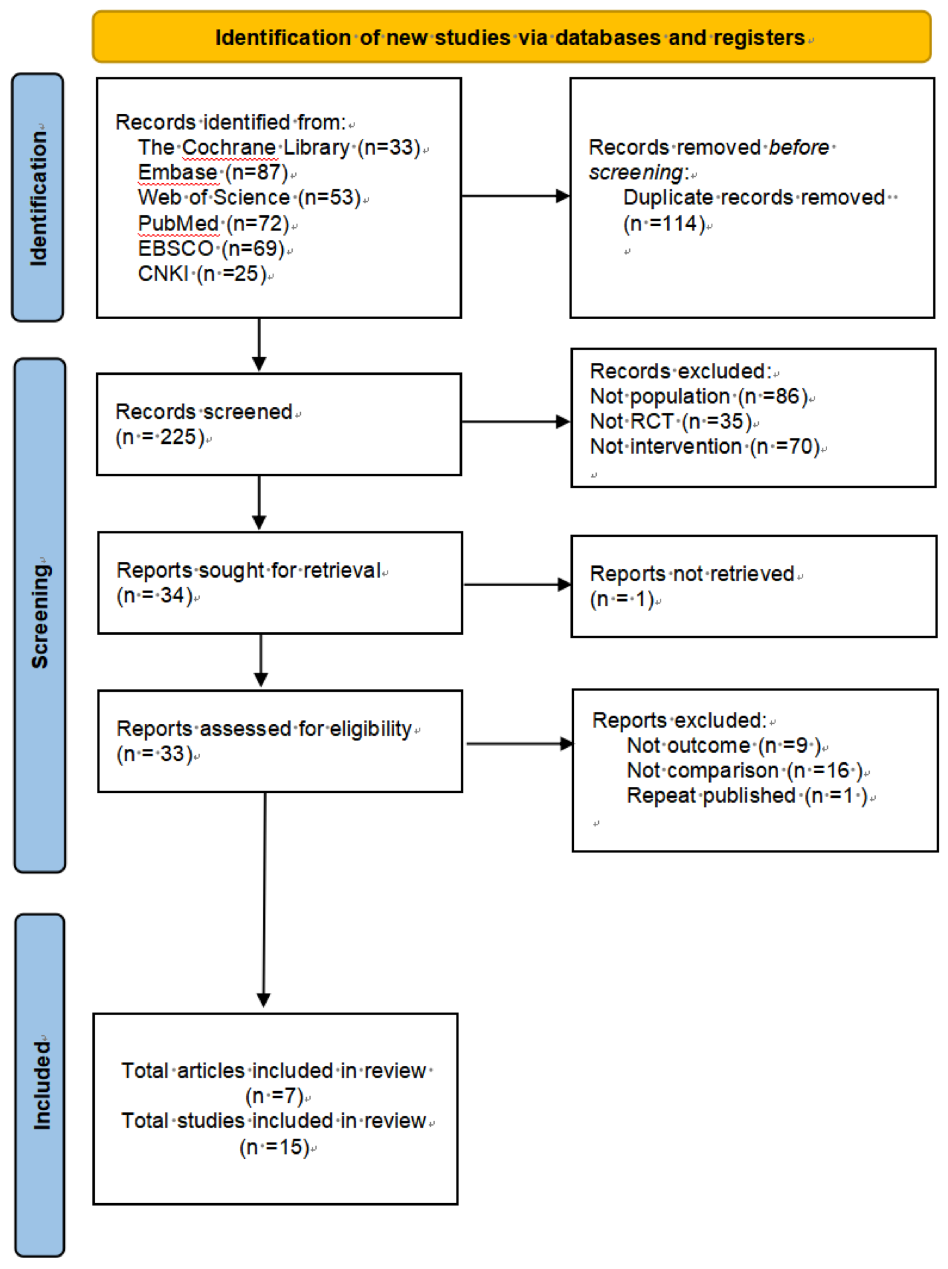
Literature search flowchart.

Ultimately, 7 high-quality studies (comprising 15 trials) were included, all being randomized controlled trials (RCTs) that primarily evaluated the intervention effects of non-invasive brain stimulation techniques [including transcranial magnetic stimulation (rTMS) and transcranial direct current stimulation (tDCS)] combined with rehabilitation approaches such as cognitive training or motor therapy in Parkinson’s disease patients. These studies spanned from 2016 to 2024, with a total sample size of 325 participants, providing a reliable data foundation for subsequent analyses (Figure 1).

### 3.2 Study selection

From the initial 339 records identified, 225 potentially eligible studies underwent full-text evaluation, with 7 studies (15 trials) ultimately meeting inclusion criteria. The screening process excluded studies based on (1) non-target populations, (2) non-RCT designs, and (3) incompatible interventions. All included RCTs evaluated combined interventions of non-invasive brain stimulation (rTMS/tDCS) with three rehabilitation approaches: cognitive rehabilitation (*n* = 4 trials), exercise rehabilitation (*n* = 5 trials), and motor-cognitive rehabilitation (*n* = 6 trials). These studies primarily assessed cognitive function (*n* = 5 studies) and emotional outcomes (*n* = 7 studies), with sample sizes ranging from 24 to 58 participants per trial (total *N* = 325). The consistent use of RCT methodology across all included studies ensured robust evidence quality for network meta-analysis (Table 1).

**Table 1:** Basic features of the included studies.

Study	Country	Intervention category	Sample size (F/M)	Age (mean ± SD)	Intervention frequency	Intervention duration
Wong 2024 (22)	China	CON	17 (8/9)	68.1 + 5.8	50 min, 2–3 times/week	5 weeks
		CR	17 (12/5)	66.8 + 6.9	50 min, 2–3 times/week	5 weeks
Pisano 2024 (23)	Italy	CON	9 (6/3)	71 ± 8.6	20 min, 5 times/week	10 days
		MCR	8 (4/4)	65.3 ± 8.5	20 min, 5 times/week	10 days
Zhang 2023 (24)	China	MR	32 (13/19)	63.87 ± 5.60	40 min, 5 times/week	8 weeks
		CR	32 (15/17)	64.03 ± 5.28	50 min, 5 times/week	8 weeks
Manenti 2018 (25)	Italy	CON	11 (6/5)	65.5 ± 6.4	50 min, 5 times/week	2 weeks
		CR	11 (4/7)	63.8 ± 7.1	50 min, 5 times/week	2 weeks
Hu 2021 (26)	China	MR	49 (19/30)	63.68 ± 5.22	45 min, 5 times/week, 1 time/day	12 weeks
		CON	49 (21/28)	64.23 ± 4.78	45 min, 5 times/week, 1 time/day	12 weeks
Luo 2019 (27)	China	CR	43 (15/28)	65.29 ± 2.50	30–45 min, once/week, 1 time/day	30 days
		CON	47 (17/26)	65.36 ± 2.41	20 min, once/day, 7 times/week	30 days
Wang 2016 (28)	China	CON	40	64.3 ± 5.45	20 min, once/day, 7 times/week	2 weeks
		CON	40	65.4 ± 5.45	60 min, once/day, 7 times/week	2 weeks
		CR	40	66.4 ± 6.45	20 min rTMS + 60 min EEG-BF, time/day, 7 times/week	2 weeks

### 3.3 Risk of bias assessment

**Figure 2:**
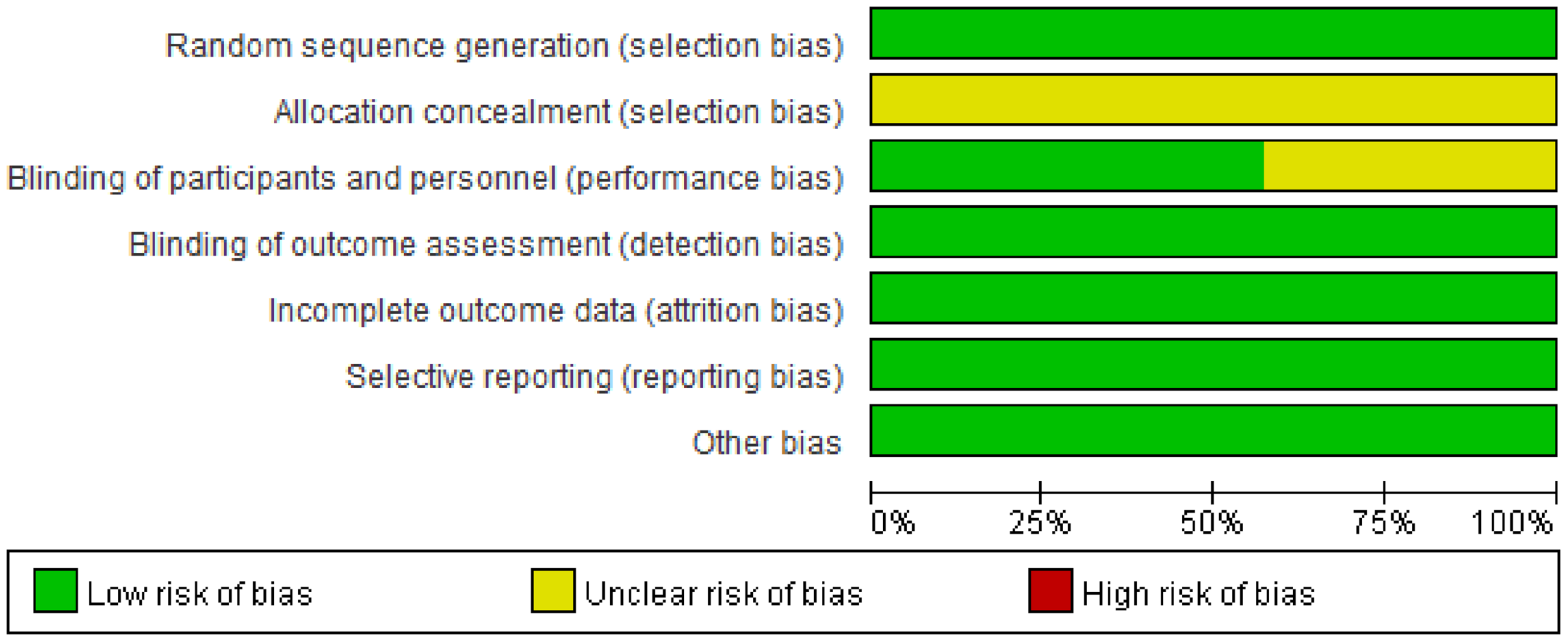
Study risk of bias in this meta-analysis.

**Figure 3:**
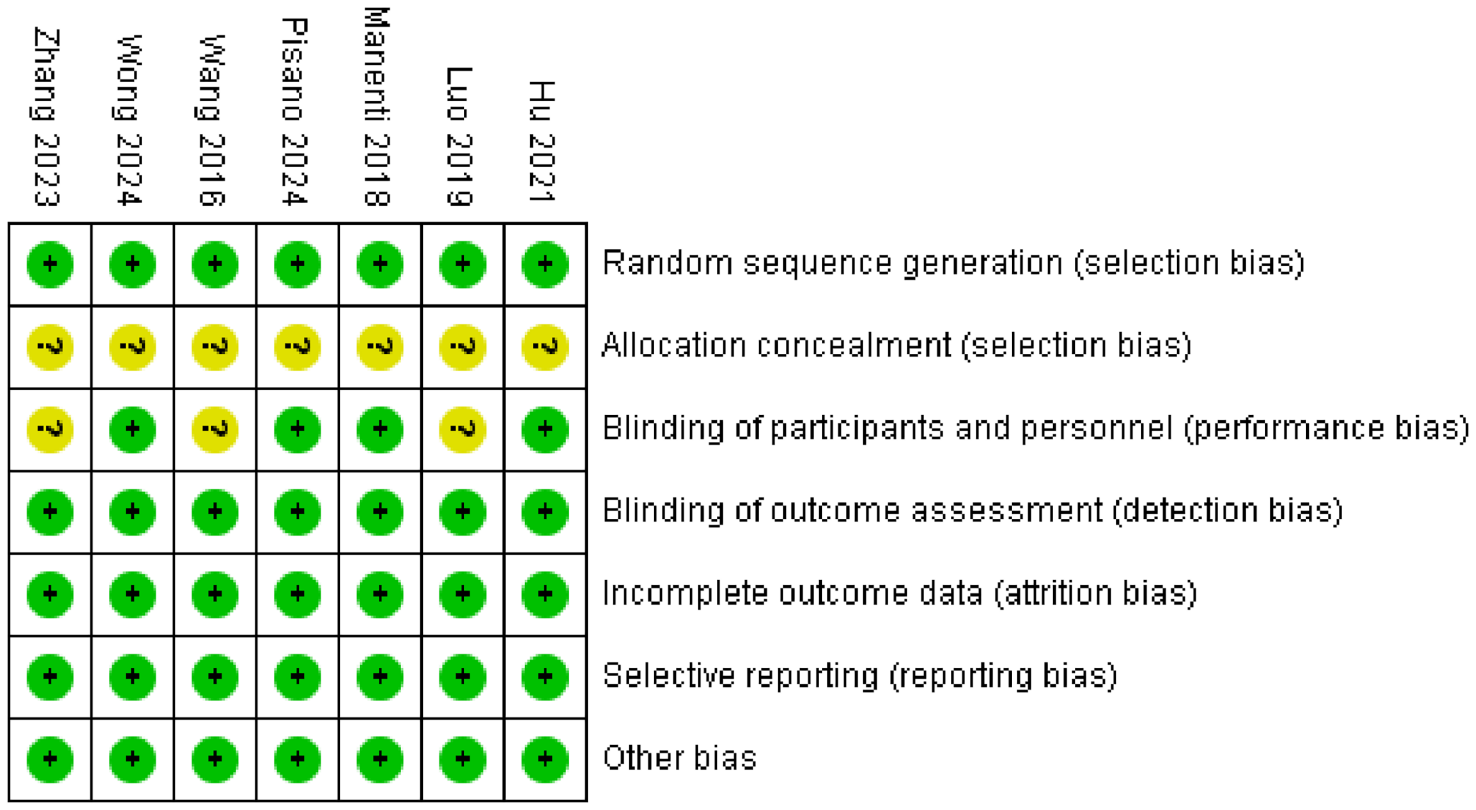
Reviewers’ judgments about each methodological quality item in the included studies. A dot with a plus sign indicates that the item was fulfilled for this study, a dot with a question mark indicates that this item was unclear in this study, and a dot with a minus sign indicates that this study did not fulfill this item.

The data (Figures 2, 3) showed that a total of 7 articles mentioned random allocation; 6 stated allocation concealment; 6 reported blinding of outcome assessment; 7 studies showed low risk of selective reporting; and 7 had no other bias. In summary, 7 articles were judged to have a low ROB.

### 3.4 Direct pairwise meta-analyses

#### 3.4.1 Primary outcomes

In terms of cognitive function improvement, CR demonstrated significant effects compared to CON (SMD = 4.88, 95% CI [−1.91, 11.67]), although with extremely high heterogeneity (I^2^ = 95%), indicating substantial between-study variability. MR showed similar significant improvement (SMD = 3.84, 95% CI [−0.18, 7.86], *p* = 0.06) but maintained considerable heterogeneity (I^2^ = 99%), while MCR did not reach statistical significance (SMD = 0.69, 95% CI [−0.02, 1.39], p = 0.06) with complete consistency across studies (I^2^ = 0%). Subgroup analysis revealed that direct comparison between CR and MR showed significant differences (SMD = 5.90, 95% CI [−2.01, 13.81], *p* = 0.14) but with unstable results due to high heterogeneity (I^2^ = 98%).

**Figure 4:**
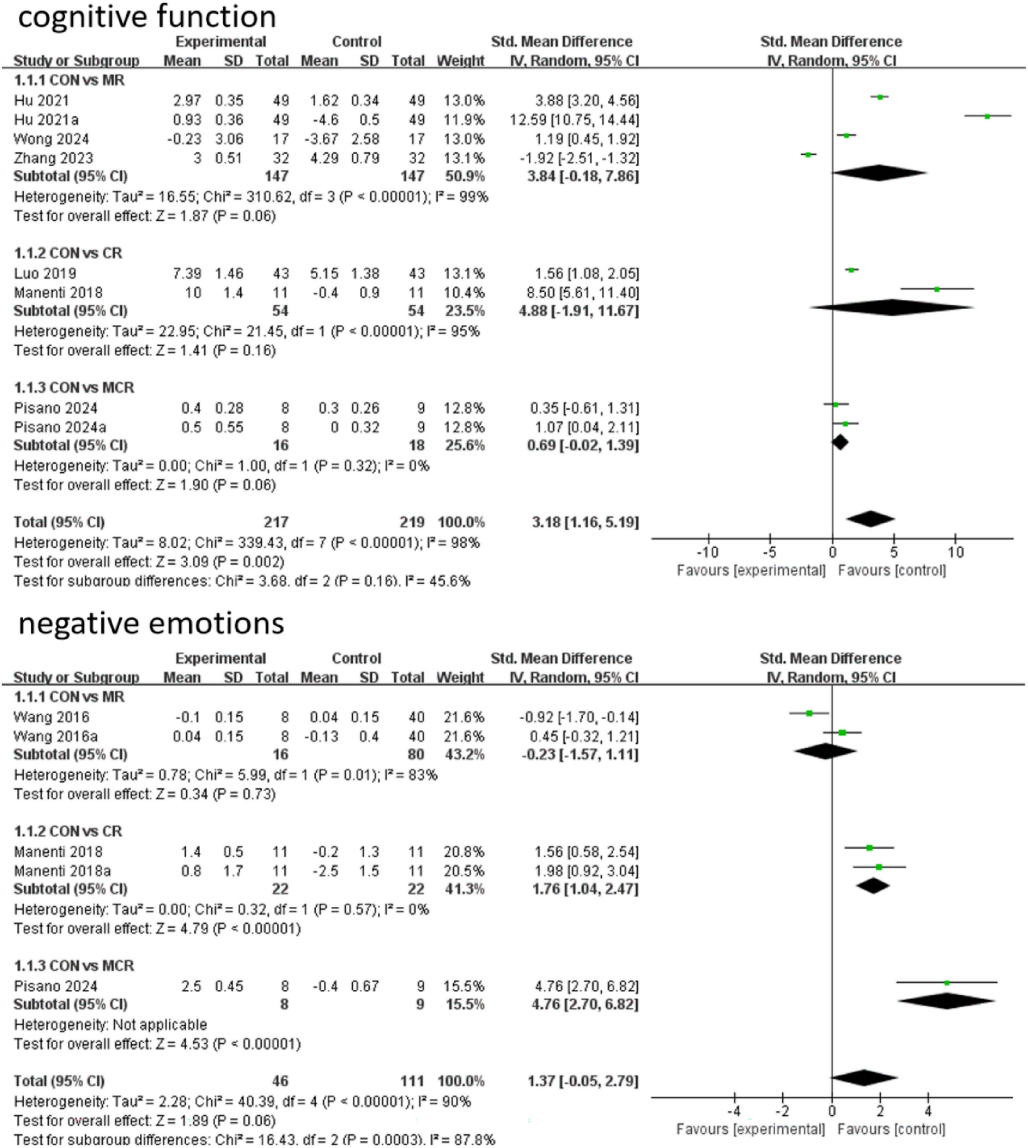
Forest plot of primary outcome.

For negative emotion outcomes, MCR displayed the most significant improvement (SMD = 4.76, 95% CI [2.70, 6.82], *p* < 0.00001) with perfect consistency across studies (I^2^ = 0%). CR also showed marked benefits (SMD = 1.76, 95% CI [1.04, 2.47], *p* < 0.00001), whereas MR did not demonstrate significant effects versus CON (SMD = −0.23, 95% CI [−1.57, 1.11], *p* = 0.73) and exhibited substantial heterogeneity (I^2^ = 83%). Notably, CR significantly outperformed MR in improving negative emotions (SMD = −2.73, 95% CI [−3.32, −2.13], *p* < 0.00001) with highly consistent results. Overall, CR showed optimal efficacy for both cognitive and emotional improvements with low between-study heterogeneity (Figure 4).

### 3.5 Network meta-analysis

#### 3.5.1 Network diagram of included studies

**Figure 5:**
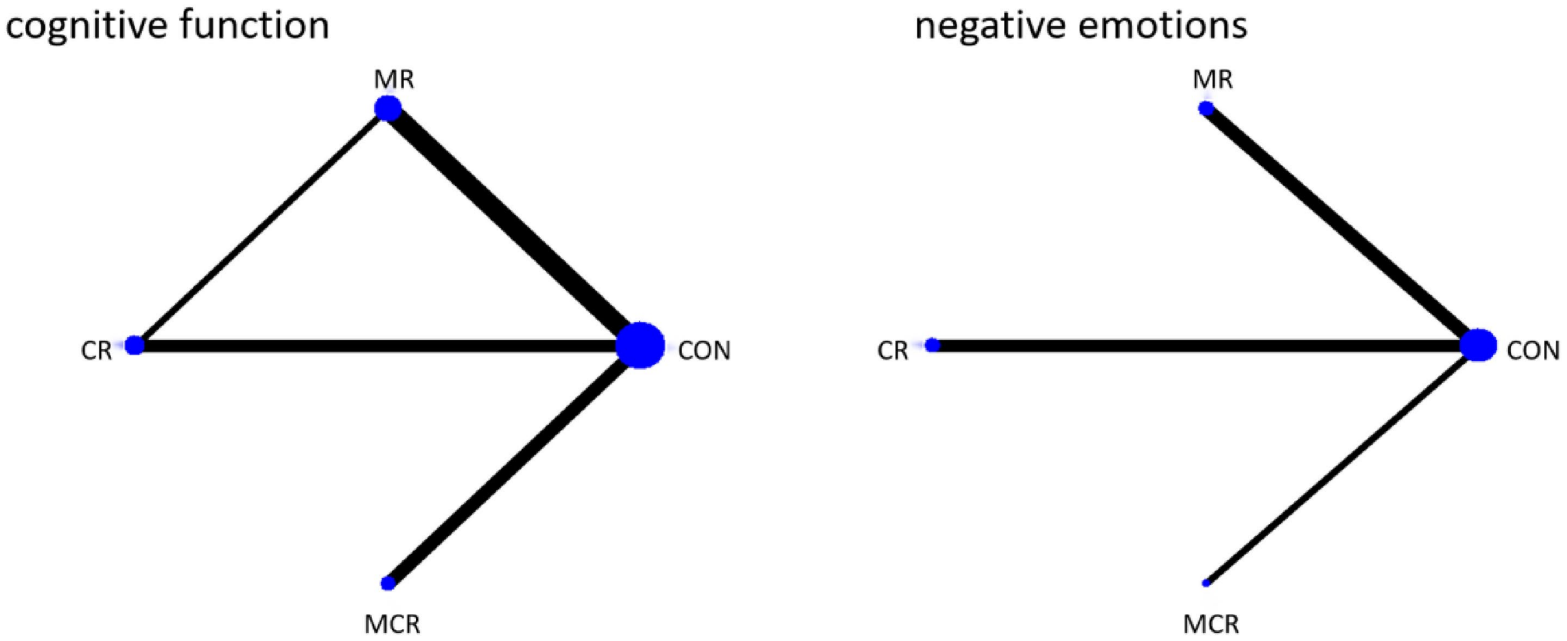
Network plot of outcome indicators.

The four dots in the diagram represent four types of interventions, the straight lines between the dots represent the existence of direct comparisons between the interventions, and the thickness of the straight lines represents the number of direct comparisons between the two types of interventions. The experimental group included non-invasive brain stimulation combined with cognitive rehabilitation, non-invasive brain stimulation combined with exercise rehabilitation, and non-invasive brain stimulation combined with motor-cognitive rehabilitation; the control group was the non-combined group, and MR was the most widely studied intervention, with fewer studies on MCR. The network diagram of the outcome metrics is detailed in Figure 5.

#### 3.5.2 Ranking of intervention effectiveness

The effectiveness of the different forms of intervention on the cognitive aspects of cognitive functioning in PD patients was ranked as CR ([SUCRA] = 81.2), showing the best intervention effect, followed by MR ([SUCRA] = 77.0), and MCR ([SUCRA] = 27.3) ranked third, both significantly better than the CON group ([SUCRA] = 14.6). This suggests that among the different forms of exercise, the CR group performed most prominently in enhancing cognitive function, followed by the MR group, while the MCR group also had some advantages, much more than the CON group, which had the most limited effect.

**Figure 6:**
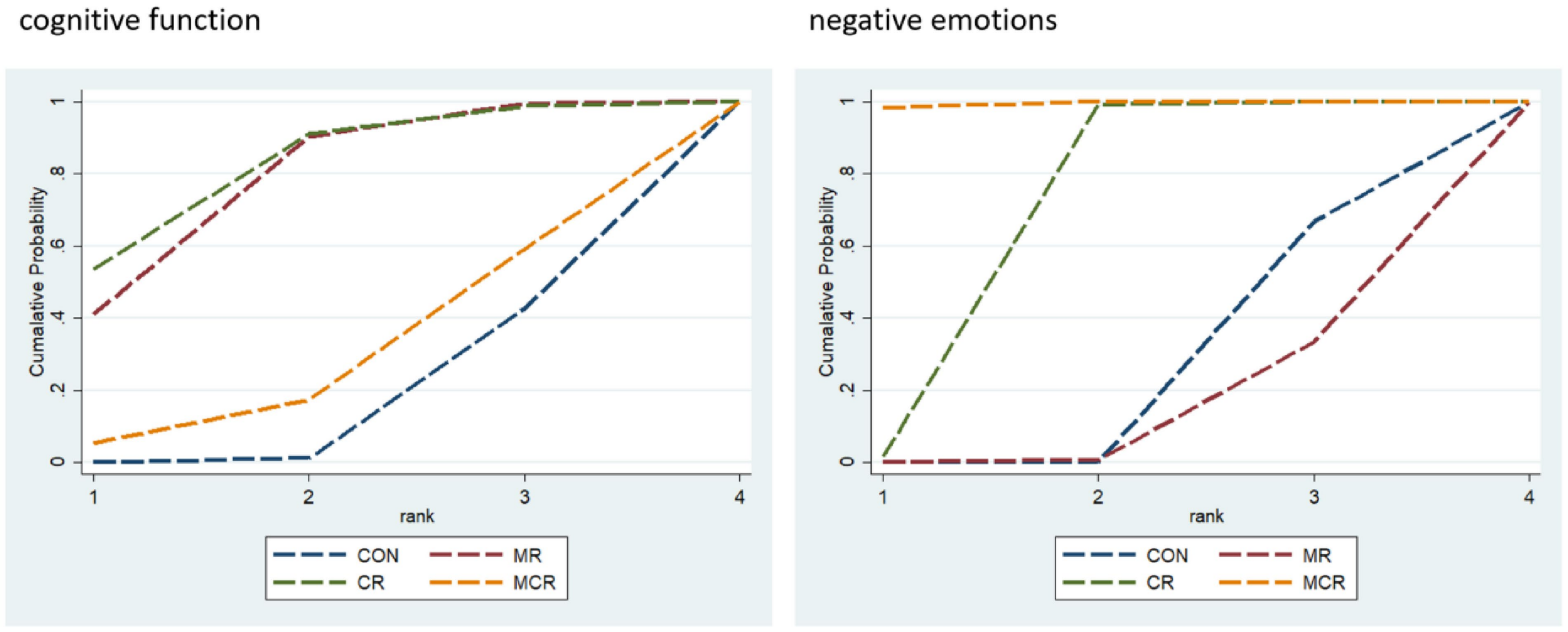
Ranking of intervention effects for outcome indicators.

The effectiveness of the different forms of intervention on the negative affective aspects of PD (Parkinson’s disease) patients was ranked as MCR ([SUCRA] = 99.5) with the best performance, followed by CR ([SUCRA] = 66.9), while MR ([SUCRA] = 11.3) ranked third, and the CON group ([SUCRA] = 22.3) was the worst performer. Based on SUCRA values and other indicators, the MCR group was significantly more effective than the other forms of intervention in reducing negative emotions, with the CR group coming in second, while the MR and CON groups had more limited effects (Figure 6; Table 2).

**Table 2:** Ranking of the probability of improving cognitive function and negative emotions.

Treatment	Cognitive function		Negative emotions	
	SUCRA (%)	Rank	SUCRA (%)	Rank
CON	14.6	4	22.3	3
MR	77.0	2	11.3	4
CR	81.2	1	66.9	2
MCR	27.3	3	99.5	1

#### 3.5.3 Summary estimates of primary outcomes

In this study, the effects of three combined intervention regimens, CR, MR, and MCR, on the improvement of cognitive function and negative emotions in PD patients were assessed by the NMA system. In terms of cognitive function improvement, each intervention regimen showed a significant effect: the CR regimen had the best effect (SMD = 4.88, 95% CI [−1.91, 11.67]), followed by MR (SMD = 3.84, 95% CI [−0.18, 7.86]), and the effect of MCR was relatively weak (SMD = 0.69, 95% CI [−0.02, 1.39]). SUCRA analysis revealed an efficacy ranking of CR (SUCRA = 81.2) > MR (SUCRA = 77.0) > MCR (SUCRA = 27.3) > CON (SUCRA = 14.6).

In terms of negative mood improvement, CR also demonstrated an optimal effect (SMD = 1.76, 95% CI [1.04, 2.47]), significantly better than the other regimens (*p* < 0.01); the MCR regimen had the next best effect (SMD = 4.76, 95% CI [2.70, 6.82]). Notably, CR showed significant advantages in both cognitive function and negative emotions; these results were visualized by ladder diagrams and SUCRA analysis plots (Table 3).

**Table 3:** Network meta-analysis matrix of outcome.

Cognitive function			
CR			
0.37 (−5.46, 6.21)	MR		
4.92 (−3.12, 12.97)	4.55 (−3.02, 12.12)	MCR	
5.63 (0.38, 10.89)	5.26 (0.76, 9.77)	0.71 (−5.38, 6.80)	CON
Negative emotions			
MCR			
3.00 (0.36, 5.63)	CR		
4.76 (2.38, 7.15)	1.77 (0.64, 2.89)	CON	
4.99 (2.40, 7.59)	2.00 (0.48, 3.51)	0.23 (−0.79, 1.25)	MR

### 3.6 Small sample effects or publication bias

**Figure 7:**
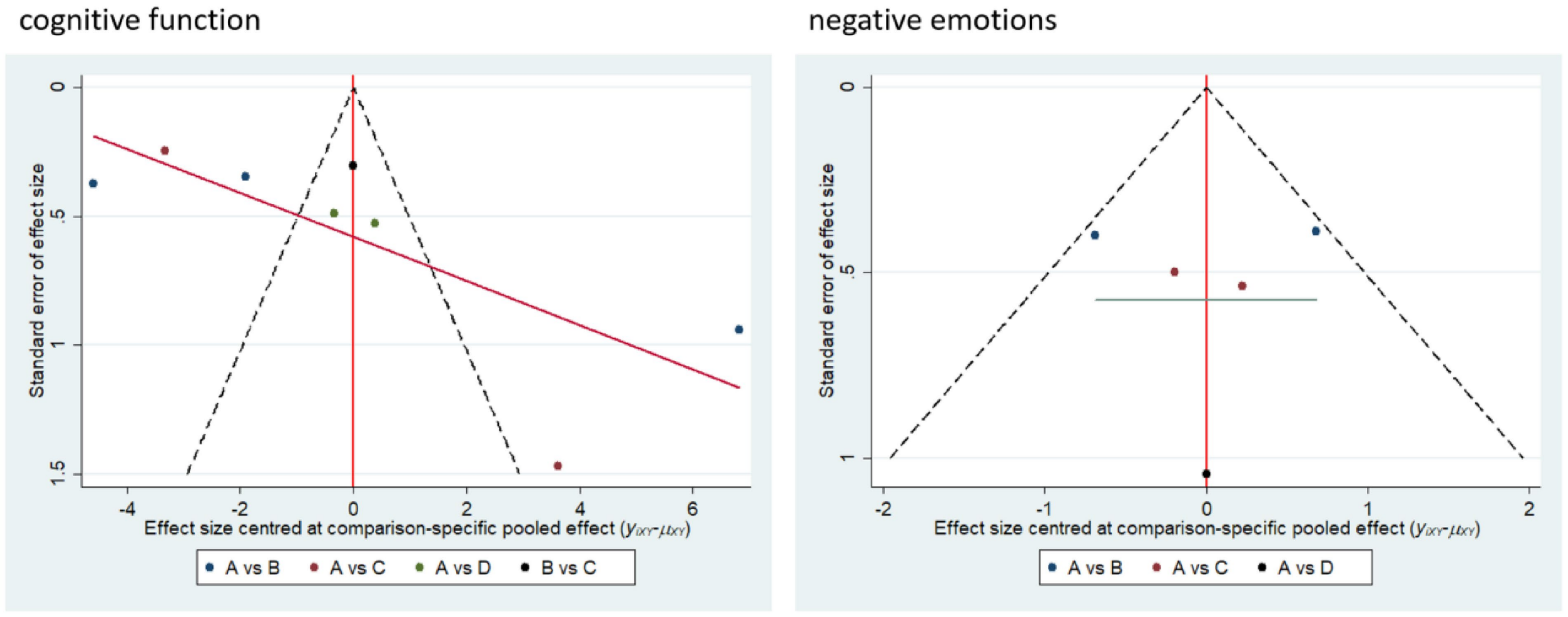
Corrected comparison funnel plot for outcome indicators.

Small sample effect estimates and publication bias tests were conducted using corrected comparison funnel plots. The results showed that the sample size of the included studies was balanced and the funnel plot was symmetrical, and no significant publication bias or small sample effects were found. This suggests that the results of the available studies have a high degree of reliability (Figure 7).

### 3.7 Sensitivity analysis

To evaluate the impact of individual studies on the pooled effect size, a sensitivity analysis was conducted. For cognitive function, after sequentially excluding each study and recalculating the pooled effect size, it was found that although there were certain fluctuations in the effect size estimates (Estimate) and 95% CI corresponding to each study, the overall trend was relatively stable. Moreover, all confidence intervals did not cross the null effect line, indicating that the pooled results of studies related to cognitive function were less affected by individual studies and had good stability.

**Figure 8:**
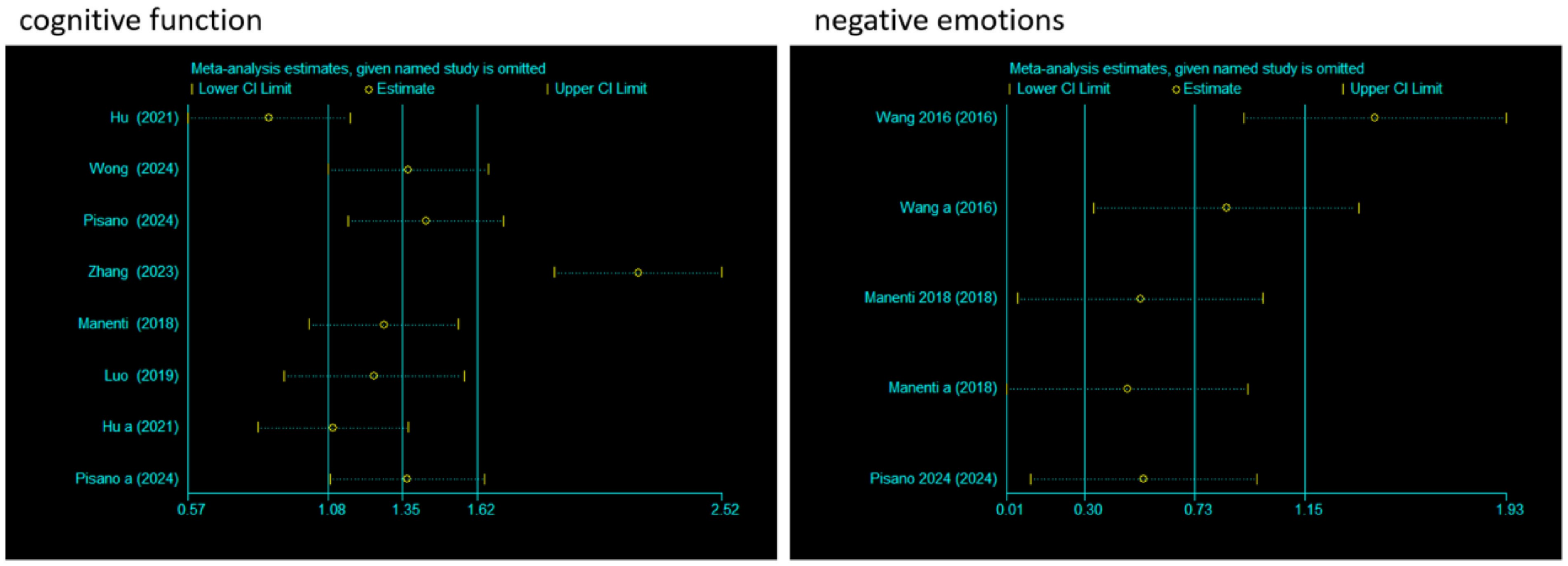
Sensitivity analysis.

For negative emotions, the operation of excluding studies one by one was also carried out. It could be seen that after different studies were excluded, the effect size estimates and confidence intervals also changed. However, there was no obvious crossing of the null effect line in each confidence interval, indicating that the pooled results of studies related to negative emotions also had good stability, and individual studies had limited interference with the overall pooled effect. In conclusion, the meta-analysis results of cognitive function and negative emotions in this study were relatively robust with low sensitivity (Figure 8).

## 4 Discussion

### 4.1 Cognitive function improvement

The results of this study showed that a non-invasive brain stimulation combined with a cognitive rehabilitation (CR) program demonstrated optimal results in improving cognitive function in Parkinson’s disease patients. This finding is consistent with the results of several studies in recent years, and a systematic evaluation by Lawrence et al. (12) indicated that targeted cognitive training combined with brain stimulation was more effective in improving cognitive deficits associated with Parkinson’s disease, particularly in executive function and working memory. This advantage may result from the synergistic effect of “dual intervention”: cognitive rehabilitation training directly targets specific cognitive dysfunctions, whereas non-invasive brain stimulation (e.g., transcranial direct current stimulation) enhances the effect of the training by modulating neuroplasticity in prefrontal cortex and basal ganglia regions (13, 14).

It is worth noting that although exercise rehabilitation combined with brain stimulation (MR) protocols was the second most effective in terms of cognitive improvement, this result is still significant. Previous studies have shown that regular exercise training can indirectly improve cognitive function (15). However, the present study found that the cognitive improvement effect of the MR program was significantly lower than that of the CR program, which may suggest that interventions directly targeting cognitive function are more effective than indirect exercise interventions for cognitive impairment in Parkinson’s disease patients.

The combined cognitive-motor therapy (MCR) program had a relatively limited performance in this study, a result that deviates from expectations. The possible reason for this is that although cognitive-motor dual-task training should theoretically produce better results, the intensity and timing of the two interventions may not be optimally synergized during actual implementation (16). In addition, patients with Parkinson’s disease may face greater challenges in allocating attentional resources when performing both cognitive and motor training, which in turn may diminish the effectiveness of the intervention (17).

### 4.2 Emotional health improvement

In terms of emotional health improvement, the findings show different patterns. The finding that the combined cognitive-motor therapy (MCR) program showed optimal results has important clinical implications. Patients with Parkinson’s disease often suffer from mood disorders such as depression and anxiety, and the results of this study suggest that interventions combining cognitive and motor components may have a significant positive impact on emotional health. This phenomenon can be explained by the theory of “cognitive-emotional interaction,” which suggests that improving cognitive function, especially executive function and emotion regulation, indirectly contributes to the improvement of emotional health (18).

A non-invasive brain stimulation combined with a cognitive rehabilitation (CR) program was the next most effective in terms of mood improvement, which is generally consistent with previous findings on the benefits of cognitive intervention for mood (19). Cognitive training can improve mood through a variety of mechanisms, including enhanced neural plasticity and improved emotion regulation capabilities (20). However, the effect of the CR program in this study was not as significant as that of the MCR program, which may suggest that integrated cognitive-motor interventions provide more comprehensive benefits for mood disorders in Parkinson’s disease patients than cognitive-focused interventions alone.

The relatively limited effect of exercise rehabilitation combined with a brain stimulation (MR) program on mood improvement is a result that deserves further investigation. Theoretically, a program combining exercise and brain stimulation should produce better mood improvement, but the actual results were less pronounced. Possible reasons for this may be that the MR program included in this study may have focused more on physiological parameters and paid insufficient attention to the emotion regulation component (21). In addition, the specific parameters of brain stimulation combined with exercise may require further optimization to maximize benefits for emotional health.

MCR shows limited cognitive benefits (SUCRA = 27.3) likely because its dual-task design splits PD patients’ limited attentional resources between motor and cognitive training, reducing cognitive stimulation specificity—unlike CR’s focused cognitive-NIBS synergy. In contrast, its top emotional efficacy (SUCRA = 99.5) comes from motor-induced endorphin release, cognitive emotion regulation, and NIBS limbic modulation, which together target PD-related mood circuit impairments more robustly than single-component interventions.

### 4.3 Clinical implications and research perspectives

This study provides valuable insights for clinical practice, but its limitations must be acknowledged. The most significant constraint is the small number of included studies (*n* = 7), which limits the statistical power of our network meta-analysis and affects the precision of effect estimates. Due to this limited evidence base, we could not adequately assess potential publication bias, and the generalizability of findings may be constrained by strict eligibility criteria and variations in intervention parameters across studies. Furthermore, the long-term sustainability of benefits remains uncertain, as most trials assessed only short- to medium-term outcomes. Future studies with larger samples, longer follow-up durations, and standardized protocols are needed to confirm these findings and enhance their clinical applicability.

## 5 Conclusion

This study demonstrates that non-invasive brain stimulation combined with cognitive rehabilitation (CR) is most effective for improving cognitive function in Parkinson’s patients (SMD = 4.88, SUCRA = 81.2), while combined motor-cognitive rehabilitation (MCR) shows superior benefits for emotional well-being (SMD = 4.76, SUCRA = 99.5). The findings support personalized treatment strategies: CR for predominant cognitive impairment and MCR for emotional symptoms. Future research should optimize intervention protocols by refining stimulation parameters, developing symptom-specific combinations, and investigating neural mechanisms through multimodal imaging. These results provide evidence-based guidance for managing Parkinson’s non-motor symptoms through targeted neuromodulation approaches.

## Data Availability

The original contributions presented in the study are included in the article/supplementary material, further inquiries can be directed to the corresponding author.
